# Evaluation of a Heart Failure Telemonitoring Program Through a Microsimulation Model: Cost-Utility Analysis

**DOI:** 10.2196/18917

**Published:** 2020-10-06

**Authors:** Chris Boodoo, Qi Zhang, Heather J Ross, Ana Carolina Alba, Audrey Laporte, Emily Seto

**Affiliations:** 1 Institute of Health Policy, Management and Evaluation Dalla Lana School of Public Health University of Toronto Toronto, ON Canada; 2 Ted Rogers Centre for Heart Research University Health Network Toronto, ON Canada; 3 Department of Medicine University of Toronto Toronto, ON Canada; 4 Peter Munk Cardiac Centre University Health Network Toronto, ON Canada; 5 Canadian Centre for Health Economics Toronto, ON Canada; 6 Centre for Global eHealth Innovation Techna Institute University Health Network Toronto, ON Canada

**Keywords:** cost utility analysis, cost effectiveness, telemedicine, heart failure, microsimulation, mobile phone

## Abstract

**Background:**

Heart failure (HF) is a major public health issue in Canada that is associated with high prevalence, morbidity, and mortality rates and high financial and social burdens. Telemonitoring (TM) has been shown to improve all-cause mortality and hospitalization rates in patients with HF. The *Medly* program is a TM intervention integrated as standard of care at a large Canadian academic hospital for ambulatory patients with HF that has been found to improve patient outcomes. However, the cost-effectiveness of the *Medly* program is yet to be determined.

**Objective:**

This study aims to conduct a cost-utility analysis of the *Medly* program compared with the standard of care for HF in Ontario, Canada, from the perspective of the public health care payer.

**Methods:**

Using a microsimulation model, individual patient data were simulated over a 25-year time horizon to compare the costs and quality-adjusted life years (QALYs) between the *Medly* program and standard care for patients with HF treated in the ambulatory care setting. Data were sourced from a *Medly* Program Evaluation study and literature to inform model parameters, such as *Medly*’s effectiveness in reducing mortality and hospitalizations, health care and intervention costs, and model transition probabilities. Scenario analyses were conducted in relation to HF severity and TM deployment models. One-way deterministic effectiveness analysis and probabilistic sensitivity analysis were performed to explore the impact on the results of uncertainty in model parameters.

**Results:**

The *Medly* program was associated with an average total cost of Can $102,508 (US $77,626) per patient and total QALYs of 5.51 per patient compared with the average cost of Can $97,497 (US $73,831) and QALYs of 4.95 per patient in the Standard Care Group. This led to an incremental cost of Can $5011 (US $3794) and incremental QALY of 0.566, resulting in an incremental cost-effectiveness ratio of Can $8850 (US $6701)/QALY. Cost-effectiveness improved in relation to patients with advanced HF and with deployment models in which patients used their own equipment. Baseline and alternative scenarios consistently showed probabilities of cost-effectiveness greater than 85% at a willingness-to-pay threshold of Can $50,000 (US $37,718). Although the results showed some sensitivity to assumptions about effectiveness parameters, the intervention was found to remain cost-effective.

**Conclusions:**

The *Medly* program for patients with HF is cost-effective compared with standard care using commonly reported willingness-to-pay thresholds. This study provides evidence for decision makers on the use of TM for HF, supports the use of a nurse-led model of TM that embeds clinically validated algorithms, and informs the use of economic modeling for future evaluations of early-stage health informatics technology.

## Introduction

### Background

Heart failure (HF) is a major public health issue with a worldwide prevalence of 26 million and 669,600 in Canada [1,2]. Half of those with a diagnosis of HF will die within 5 years, and up to 80% will die within 10 years [3-5]. Furthermore, flare-ups of HF symptoms occur often and can result in frequent hospitalizations, with more than 50% of individuals being readmitted within 6 months of discharge [6,7]. Reasons for rehospitalizations include incomplete treatment in hospitals, poor coordination of services or poor communication of care plans at discharge, inadequate access to services, poor patient education, failure to optimize therapies, and lack of long-term monitoring for early signs of worsening health [7]. HF-associated hospital admissions have been estimated to cost the Canadian health care system Can $482 (US $364) million in 2013, and the cost is expected to increase to Can $720 (US $543.14) million by 2030 [8].

It has been recommended that disease management interventions that enable patient empowerment, education, and clinical follow-up should be integrated within the system of care for patients with HF because these interventions have been associated with reduced hospitalization rates and improved quality of life (QoL) and survival [9]. In response, the *Medly* telemonitoring (TM) system and program, which was deployed to augment the existing standard of care at the Ted Rogers Centre for Heart Research at the University Health Network (UHN), was designed to shift traditional episodic care of HF to a more continuous paradigm where care is extended into the daily lives of patients rather than confined to health care institutions. This program enables patients to record symptoms and physiological measurements, including weight, blood pressure, and heart rate, which are then transmitted to a registered nurse coordinator who reviews and responds to alerts and serves as the first resource for patients. Overall, meta-analyses have shown that TM programs similar to *Medly* reduce all-cause mortality and hospitalizations when compared with the standard of care without TM [10-14]. However, other studies have also shown null or mixed results for TM [15-18]. Some of this uncertainty in effectiveness can be attributed to the heterogeneity of the studies, such as patient demographics, characteristic differences between the evaluated interventions, and quality of the trial [19].

The decision to implement interventions are often dependent on cost-effectiveness. However, there is a lack of economic evidence for TM stemming from the challenges associated with conducting economic evaluations owing to the heterogeneity and complexity of TM [20,21]. Specifically, diversity stemming from clinical conditions under study, technology, applications, objectives, and context makes comparisons between telemedicine interventions difficult [21]. That said, a number of studies have been conducted that included evaluations of the financial impact of TM for patients with HF, with many reporting savings. However, most studies did not conduct a full economic evaluation [22-33], such as a cost-effectiveness or cost-utility analysis (CUA) [34]. Studies that included a full economic analysis did not evaluate the long-term effects of TM because time horizons of 18 months or less were used [35-37], or the studies were conducted outside of Canada [38-40]. This is owing to the lack of long-term Canadian economic evaluations of TM interventions for patients with HF, information on the life expectancy of this patient population, the possible fluctuations of health status over time, and the nuances of the Canadian population and health care system.

### Study Objectives

The objective of this study was to evaluate the long-term cost-effectiveness of TM for patients with HF within a Canadian context from a public health care payer perspective, referencing costing data and concepts from a TM program, *Medly*, implemented at a large academic hospital in Ontario, and data from the literature. Specifically, the central research question is as follows: What is the cost utility of the *Medly* program for patients with HF compared with the standard of care in Ontario? This question will be explored through the application of a microsimulation model.

## Methods

The methods used in this study follow the Consolidated Health Economic Evaluation Reporting Standards [41].

### Type of Economic Evaluation

A CUA was performed. A CUA was chosen because it allows for the effectiveness outcome to be comparable with that of other disease groups and across interventions, making it the gold standard for economic evaluations. Furthermore, there is utility evidence available for patients with HF, allowing for the use of quality-adjusted life years (QALYs) [34].

### Target Population

The target population was a cohort of ambulatory patients with HF from the UHN in Toronto, Canada, enrolled in the *Medly Program Evaluation* study. Table 1 presents information on the baseline patient characteristics and the missing data. Owing to the limitations associated with manual extraction of data from patients’ clinical notes and inconsistent laboratory testing orders, there were considerable missing data. The nature of the missingness was not quantitatively analyzed, and it was assumed that the missing data were randomly distributed. The data reported correspond to the set of variables that were used in the Seattle Heart Failure Model (SHFM), which is a multivariate Cox hazard model used to predict mortality [42-45]. As of June 31, 2019, 315 patients were enrolled in the program, based on a joint decision between the patient and the cardiologist at either a follow-up outpatient visit or after an inpatient hospital stay.

**Table 1:** Baseline patient characteristics of the Medly Program Evaluation cohort (n=315; number of patients unless specified otherwise).

Characteristics	Overall	Missing, n
Age (years), mean (SD)	58.23 (15.43)	2
Proportion of females, n (%)	69 (22.0)	2
Proportion of ischemic etiology, n (%)	65 (28.5)	87
Proportion of beta blockers, n (%)	270 (89.4)	13
Proportion of aldosterone blockers, n (%)	215 (71.2)	13
Proportion of ARBs^a^, n (%)	82 (27.2)	13
Proportion of ACE^b^ inhibitors, n (%)	137 (45.5)	13
Proportion of allopurinol, n (%)	41 (13.6)	13
Percentage LVEF^c^, mean (SD)	32.07 (13.62)	7
New York Health Association (average class), mean (SD)	2.36 (0.59)	13
Systolic blood pressure (mm Hg), mean (SD)	110.36 (17.91)	53
Percentage of lymphocytes, mean (SD)	22.18 (9.07)	52
Sodium (mEq/L), mean (SD)	137.73 (3.06)	33
Cholesterol (mg/dL), mean (SD)	154.77 (52.71)	83
Hemoglobin (g/dL), mean (SD)	13.33 (1.99)	52
Urate (mg/dL), mean (SD)	7.97 (2.70)	86
Weight (kg), mean (SD)	83.39 (20.04)	44
Furosemide-equivalent dose (mg/day), mean (SD)	99.57 (123.93)	16
Proportion of implantable cardioverter-defibrillators, n (%)	165 (56.5)	23

^a^ARB: angiotensin II receptor blocker.

^b^ACE: angiotensin-converting-enzyme.

^c^LVEF: left ventricular ejection fraction.

### The Intervention-Medly

In August 2016, the *Medly* program was deployed to augment the existing standard of care at the Ted Rogers Centre for Heart Research at the UHN. Patients are trained to use the technology, and the importance of taking daily readings is emphasized. Patients then use the intervention as long as there are clinical benefits as determined by both the clinician and the patient. The program is led by a registered nurse coordinator who reviews and responds to alerts and serves as the first resource for patients’ clinical concerns or technical troubleshooting.

**Figure 1:**
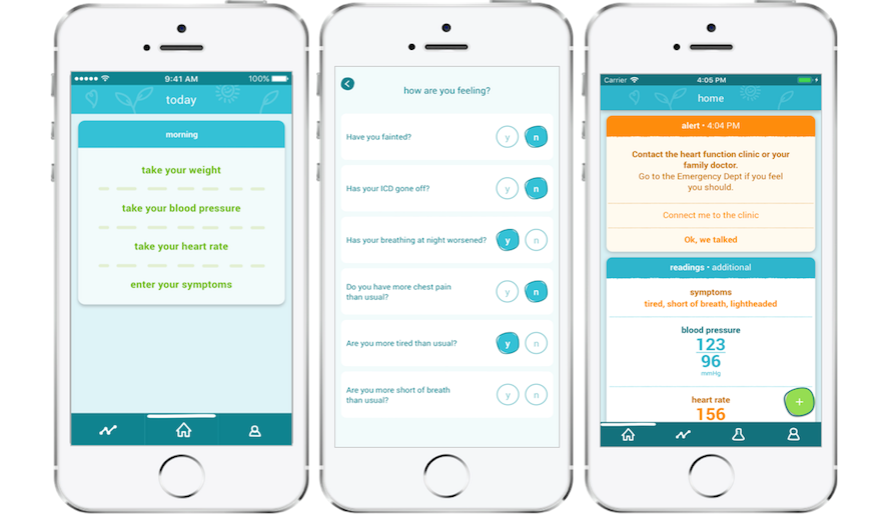
Medly app showing instructions for required readings (screen 1), the symptoms questionnaire (screen 2), and personalized self-care feedback (screen 3).

The main component of the program is the *Medly* smartphone app. Patients use the app to record their body weight, blood pressure, and heart rate and to answer a short yes or no questionnaire about their symptoms. Patients are asked to take these readings daily immediately after they wake up. These data are then processed by a clinically validated algorithm to interpret the readings relative to the patient’s target thresholds set by the most responsible HF physician [46]. If the algorithm determines that the recordings are within the target range, patients are presented with a prompt stating their HF is in a stable condition. If the algorithm deems that the readings are outside the target range and/or identify an abnormal trend in weight gain, the patient is prompted with self-care feedback such as taking an additional dose of their diuretic medication and to contact their care provider or to visit the emergency department (ED). Figure 1 shows screenshots of some of the various interfaces with which patients interact. In addition to the self-care messages, the registered nurse coordinator receives the alert via email and triages the event. The nurse also responds to technical troubleshooting. A full-time nurse is projected to be able to manage approximately 500 patients through the *Medly* program. Alerts and all patient TM data can be viewed on the *Medly* clinical dashboard. Other features of the *Medly* app include graphical trends of specific measurements and an automated phone call to remind patients to take their daily measurements if it is past 10 AM (can be disabled per patient’s request).

### Key Data Source

This study referenced the ongoing *Medly* program evaluation, which included multiple pre- and posttest analyses on patient-level impacts, patient adherence, and cost. Quantitative data analyses leverage data that were collected at the 6-month follow-up as a part of the standard of care, such as health care utilization and laboratory results from electronic patient records, while also using data from the TM system. The results of this study have been published in the study by Ware et al (2020) [47], and further details on the study can also be found therein.

### Comparators

In this analysis, the intervention group was a cohort of patients with HF enrolled in the *Medly* program. The comparator group consisted of patients with HF who received standard care, which does not include the use of TM. It was assumed that standard care was conducted according to the typical care practices in Ontario, which involves specialized multidisciplinary HF clinics, although care models may vary among clinics [48].

### Perspective

This analysis was conducted from the perspective of the public payer, namely the Ontario Ministry of Health because Medly is currently implemented in a publicly funded health care system.

### Time Horizon and Discounting

A time horizon of 25 years was adopted to determine the long-term cost and outcomes associated with *Medly* for the patient population with HF. Costs and outcomes were discounted at an annual rate of 1.5%, as recommended by the Canadian Agency for Drugs and Technologies in Health [49].

### Model Framework, Conceptualization, and Technique

All analysis and model construction were conducted using RStudio [50]. The model consists of 7 mutually exclusive states: 4 states specific to HF severity, 2 states for hospitalization events, and 1 absorbing state for death. Patients with HF can alternate between a state of decompensation (or symptom exacerbation or functional capacity impairment) and a state of clinical stability. To capture this, the model was stratified by, and allowed for, transitions between New York Health Association (NYHA) functional classes. The NYHA functional classification is a common measure used by clinicians to classify the severity of symptoms in patients with HF, in which a higher class indicates worse health [51]. Hospitalizations mark a fundamental change in the natural history of HF with subsequent increased rehospitalizations and higher mortality rates as the patient’s disease progresses [52,53]. A cycle length of 1 month was chosen to account for 30-day readmission rates common in the HF population [54]. As recommended by Naimark et al [55] for models that are relatively simple and have a cycle length of a month or less, a half-cycle correction was omitted.

**Figure 2:**
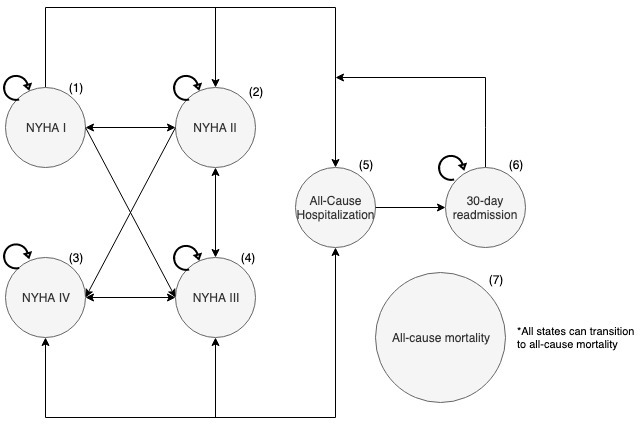
Conceptual representation of the microsimulation model structure. States 1 to 4 represent the transitions between New York Health Association classes. States 5 and 6 show transitions into and between hospitalization states. State 7 is an absorbing state representing death, where all states can transition to. NYHA: New York Health Association.

The modeling technique chosen was a patient-level state-transition model, also known as a first-order Monte Carlo microsimulation. This model was appropriate because it can capture patient heterogeneity that is common in patients with HF and is also the preferred option for modeling chronic disease [56]. Figure 2 shows the conceptualization of the Markov model that was developed to represent an individual’s progression of HF.

### Parameter Estimates

The values used in the model were based on a literature review. The values inputted into the model are conditional on patient characteristics. Patients with a more limited functional capacity by NYHA class have a higher risk of hospitalization [57-61]. In addition, the risk for readmission is highest within 30 days of hospital discharge [52]. As a patient’s NYHA functional class can change over time, the probability of transitioning between classes was derived from the large-scale international SENIORS study [62,63]. All-cause mortality during hospitalization was based on the study by Yeung et al (2012) [64]. The SHFM was used to derive a survival curve for each patient over their lifetime, which is a multivariate Cox hazard model that has been validated on multiple cohorts of patients with HF [42-45].

### Generating Virtual Patient Profiles

To generate virtual patient profiles, a Cholesky decomposition was performed on a correlation matrix that describes the interdependence between patient characteristics [56]. The matrix was derived from a consolidated representative sample of 7125 patients with HF from the University of Washington, Prospective Randomized Amlodipine Survival Evaluation, Valsartan Heart Failure Trial, and Italian Hearty Failure Registry [65] (Multimedia Appendix 1). Values for each patient characteristic were sampled from a multivariate normal distribution, defined by the *Medly Program Evaluation* cohort’s mean and SD in Table 1 using the R-package “PoisBinOrdNonNor” [66,67]. Patient characteristics included clinical, pharmacological, device, and laboratory data, based on the SHFM developed by Levy et al (2006) [43].

### Effectiveness

The 2 primary outcomes that TM interventions for patients with HF aim to improve are all-cause mortality and all-cause hospitalization rates. The risk of all-cause hospitalization was based on evidence from the *Medly* program evaluation, which reported a relative risk (RR) of 0.753 (95% CI 0.634-0.879) for patients using *Medly* (Multimedia Appendix 1; All-cause hospitalization). Owing to the lack of an interdependent comparative group and small sample size of the *Medly* program evaluation, it was not possible to evaluate its effectiveness in reducing mortality. Therefore, the estimate was based on a meta-analysis of the effectiveness of TM for patients with HF by Yun et al (2018) [11]. This meta-analysis only included randomized controlled trials that defined TM as the transmission of biological information, such as body weight, heart rate, and blood pressure via telecommunication technologies. Owing to this strict inclusion criteria, it was deemed comparable evidence for the expected benefits that Medly users could experience. It was reported that TM users had an RR of 0.81 (0.70-0.94) for all-cause mortality compared with 416 of 3724 patients in the TM group to 483 of 3733 patients in the control group [11]. The study follow-up periods ranged from 3 months to 15 months, with 1 study having a 4 year follow-up. The meta-analysis also presented a subgroup analysis of only asynchronous interventions (ie, removal of real-time and teleconferencing technologies), where an RR of 0.79 (0.66-0.94) was reported for all-cause mortality, which is similar to the primary analysis.

### Measurement and Evaluation of Health

Each state in the model has an associated utility value between 0 and 1. Utility values for each health state were derived on the basis of values from other health economic evaluations of HF interventions. NYHA classes are commonly used to categorize patients with HF based on severity of symptoms, and studies have estimated utility values for each class [60,62,68,69]. All utilities and the sources of the values are presented in Table 2. To adjust for the decrease in the QoL patients experience when hospitalized [70], the patient utility value in the model was decreased by 0.059 in the hospitalization state, consistent with Sandhu et al (2016) [71].

**Table 2:** Model parameters conditional on New York Health Association (NYHA) class including living with heart failure costs (in Can currency), utilities, probability of hospitalization, and transitions between NYHA classes.

Description		NYHA^a^ I	NYHA II	NYHA III	NYHA IV^b^	Source	Distribution
**Health care costs**							
	Emergency department costs, Can $ (US $)	0.00	0.00	62.83 (47.20)	62.83 (47.20)	Medly Program Evaluation	Gamma
	General practitioner visit costs, Can $ (US $)	0.00	0.00	12.87 (9.67)	12.87 (9.67)	Medly Program Evaluation	Fixed
	Drug costs (only if patient age is ≥65), Can $ (US $)	52.00 (39.06)	52.00 (39.06)	79.43 (59.67)	208.16 (156.38)	Kaul et al (2011) [72]	Gamma
	Outpatient costs, Can $ (US $)	97.00 (72.87)	97.00 (72.87)	97.00 (72.87)	97.00 (72.87)	Medly Program Evaluation	Gamma
	Total monthly cost of living with heart failure, Can $ (US $)	149.00 (111.94)	149.00 (111.94)	252.13 (189.42)	380.86 (286.14)	OCCI^c^[73], SOB^d^ [74], Kaul et al (2011) [72]	N/A^e^
**Utilities (range)**							
	Living with heart failure	0.81 (0.81-0.90)	0.72 (0.72-0.83)	0.59 (0.59-0.74)	0.508 (0.508-0.59)	Yao et al (2008) [62]	Beta
	Probability of all-cause hospitalization	0.0152 (0.008-0.023)	0.024 (0.012-0.036)	0.024 (0.012-0.036)	0.154 (0.077-0.231)	Ford et al (2012) [68], Borisenko et al (2015) [69]	Beta
**Transition probabilities between NYHA classes** **(probability)**							
	NYHA I	0.977	0.019	0.004	0	Flather et al (2005) [63], Yao et al (2008) [62]	Dirichlet
	NYHA II	0.008	0.981	0.01	0.001	Flather et al (2005) [63], Yao et al (2008) [62]	Dirichlet
	NYHA III	0	0.034	0.96	0.006	Flather et al (2005) [63], Yao et al (2008) [62]	Dirichlet
	NYHA IV	0	0	0.055	0.945	Flather et al (2005) [63], Yao et al (2008) [62]	Dirichlet

^a^NYHA: New York Health Association.

^b^NYHA IV assumed same as NYHA III, except drug cost.

^c^OCCI: Ontario Case Costing Initiative.

^d^SOB: Schedule of Benefits for Physician Services.

^e^N/A: not applicable.

### Resource Use and Costs

Health care utilization was based on data from the *Medly* program evaluation, and hospitalization and ED visit costs were derived from the Ontario Case Costing Initiative (OCCI) report for 2017-2018 using diagnosis codes I500, I509, and I501 [73]. The unit cost per outpatient visit was based on a paper outlining health care utilization for patients with HF over the last 6 months of their lives by Kaul et al (2006) [72] in a comparable health care system in Alberta. This was based on the provincial ambulatory care case mix group, which captures direct and indirect functional center costs [75]. Physician fees for general practitioner (GP) visits were based on billing code A005 in Ontario’s Schedule of Benefits [74]. The unit costs were multiplied by utilization data from the *Medly* program evaluation to calculate the monthly costs (Table 3). Median values for utilization were used because the distribution of health care utilization is typically left-skewed [76]. Monthly drug costs were incurred on the part of the public payer under the Ontario Drug Benefit program for patients aged 65 years and older. The monthly drug costs were calculated on the basis of the costs reported in Kaul et al (2006).

Of note, the number of outpatient visits for both the intervention and comparator-simulated cohorts was limited to those that occurred at UHN because information outside of UHN’s services was unavailable at the time of the study. Furthermore, self-reported ED visits were used because UHN patient records may underreport the true number of ED visits as patients may live at some distance from UHN and may instead visit a community hospital for an emergency. Self-reported GP visits were used because UHN data do not record this information.

**Table 3:** Median health care utilization over 6 months before using Medly, unit costs per service (in Can currency), and associated distribution stratified by New York Health Association (NYHA) classes. N is the number of patients in each NYHA class.

Type of resource	Unit cost, mean (SD), Can $	Source for unit cost	NYHA I health care utilization (n=44)	NYHA II health care utilization (n=166)	NYHA III health care utilization (n=93)	NYHA IV health care utilization (n=1)	Distribution
Emergency department (self-reported)	377.00 (374.00)	Ontario Case Costing Initiative	0	0	1	—^a^	Negative binomial
Outpatient visit	291.33 (161.11)	Kaul et al (2011) [72]	2	1	2	—	Negative binomial
General practitioner visit (self-reported)	77.20	Schedule of Benefits	0	1	2	—	Negative binomial
Drug costs over 6 months	1248.96 (2233.52)	Kaul et al (2011) [72]	—	—	—	—	Gamma

^a^No data available.

### Medly Costs and Deployment Models

Costs related to implementation and maintenance of *Medly* were provided by the *Medly* project management and development team (Table 4). The fixed costs associated with implementation were based on a system that delivers care to 1000 patients. The operational cost per patient included costs associated with asset management (technical and application support) and on-site frontline support for patients and clinicians, which was delivered via 2 registered nurse coordinators hired by the *Medly* program. Two registered nurses were included according to the *Medly* project management team’s cost projections for 1000 patients. This is similar to what was reported by Ware et al (2020) [47], where approximately 300 patients were managed by 1 registered nurse coordinator. The variable cost per patient included the cost of the device and equipment, depending on the equipment that was loaned to the patient. The costs of the device and the equipment were based on a rental model. These costs were adjusted for monthly costs according to the cycle length of the model.

The variable cost was based on a mix of models where users can fall into 1 of the 3 categories: Full Kit (FK), Bring Your Own Phone (BYOP), and Bring Your Own Everything (BYOE). An FK user refers to a user who was provided with all necessary equipment for the technology, which is currently funded by the *Medly* program, including a smartphone with a data plan, blood pressure monitor, weight scale, and licensing fee. A BYOP user brings their own phone and pays for their own data plan, but the blood pressure monitor, weight scale, and *licensing fee are provided by the Medly* program. The BYOE user brings their own equipment and is provided with just the licensing fee by the program. The reference case analysis uses a ratio of 2 FK:1 BYOP:2 BYOE, which was based on the number of each category of users in *Medly*’s current implementation. All costs were converted to 2019 Canadian dollars using Statistics Canada’s Consumer Price Index to adjust for inflation [77].

**Table 4:** Parameter estimates not conditional on the New York Health Association class including hospitalization costs (in Can currency) and disutility, readmission rates, Medly costs (in Can currency), and Medly effectiveness estimates.

Parameters		Value	Source	Distribution
**Costs**				
	Hospitalization cost per admission (Can $), mean (SD)	8908 (16,867)	OCCI^a^	Gamma
	Hospitalization length of stay days, mean (SD)	5.9 (11.2)	OCCI	Log normal
	Medly fixed costs for site implementation	102,500	Medly program	Fixed
	Medly operational cost per patient (cost per month), Can $	44.67	Medly program	Fixed
	Medly Full Kit cost per patient (cost per month), Can $	67.56	Medly program	Fixed
	Medly Bring-Your-Own-Phone cost per patient (cost per month), Can $	18.87	Medly program	Fixed
	Medly Bring-Your-Own-Everything cost per patient (cost per month), Can $	3.80	Medly program	Fixed
**Hospitalization,** **probability**				
	30-day readmission probability (95% CI)	0.159 (0.089-0.159)	Yeung et al (2012) [64]	Beta
	Disutility for hospitalization (95% CI)	0.059 (0-0.11)	Sandhu et al (2015) [71]	Beta
**Medly treatment effect,** **disutility**				
	RR^b^ for hospitalization	0.857 (0.703-1.014)	Medly	Log normal
	RR for morality	0.81 (0.70-0.94)	Yun et al (2018) [13]	Log normal

^a^OCCI: Ontario Case Costing Initiative.

^b^RR: relative risk.

### Reference Case Analysis

The expected values for all model parameters were used for the deterministic analysis. The cohort size was assumed to be 1000 patients. Each simulated patient progressed through the model twice until death; once as a patient using *Medly* and again as a patient receiving standard care. Each patient incurred costs and QALYs depending on the health state they were in. Total costs and QALYs were summed for both the *Medly* simulations and standard care simulations. From this, the average incremental cost-effectiveness ratio (ICER) per patient was computed using the following formula:


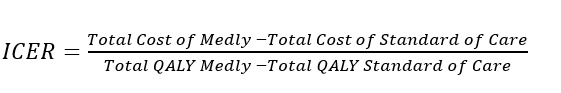


Monte Carlo standard errors were also reported to show how the results vary owing to patient heterogeneity and randomness introduced from patients transitioning to each state.

A second-order probabilistic analysis was also conducted to characterize the uncertainty in the deterministic results. Each parameter in the model was assigned a distribution based on the nature of the input parameter [56]. R-package “fitdistrplus” was used to fit negative binomial distributions for the health care utilization data from the *Medly* program evaluation via maximum likelihood estimation [78]. Details regarding how distributions were chosen based on Akaike Information Criterion and Bayesian Information Criterion scores are available in Multimedia Appendix 1 ([11,54]; Curve fitting). Values were then randomly selected from the respective distributions and assigned as the input parameter. This process was repeated 1000 times. The results for each iteration were plotted on a cost-utility plane to visualize whether *Medly* was cost-effective, cost-saving, cheaper, or dominated. The simulations were also plotted onto a cost-effectiveness acceptability curve (CEAC), where the proportion of simulations that resulted in an ICER under a range of willingness-to-pay (WTP) thresholds are plotted. A commonly used WTP threshold is Can $50,000 (US $37,718)/QALY [79,80].

### Scenario Analyses

#### NYHA Functional Classes

Currently, most patients enrolled in the *Medly* program have HF that corresponds to NYHA functional classes II or III. For this scenario analysis, cohorts were generated for NYHA functional classes I, II, and III and simulated deterministically and probabilistically. NYHA functional class IV was not included because only 1 functional class IV patient was enrolled in the *Medly* program. Average ICERs per NYHA functional class were calculated in both the deterministic and probabilistic models, and CEAC curves were produced on a plot to visualize for which classes *Medly* was most likely to be cost-effective.

#### Deployment Model

As mentioned, the *Medly* program currently offers 3 types of kits where the ratio of types of user is 2:1:2 for FK, BYOP, and BYOE, respectively. As the *Medly* program expands, understanding how the ICER changes when costs are shifted from the public dollar to the individual is informative to decision makers. Therefore, this analysis explored various proportions of user types. Specifically, each patient in the reference case cohort was randomly assigned FK, BYOP, and BYOE according to predefined ratios. The ratios of interest were 1:0:0 (100% FK), 1:4:5 (40% BYOP, 50% BYOE), and 0:0:1 (100% BYOE). These were identified as all FK, mixed deployment, and all BYOE, respectively.

### Effectiveness

One-way deterministic analyses were also conducted to address the uncertainty associated with the estimates for *Medly*’s effectiveness in reducing all-cause hospitalizations (RR 0.63-0.88) and mortality (RR 0.70-0.94). Specifically, 95% CI for each estimate were inputted into the model, and the range of ICERs was presented.

## Results

### Reference Case Analysis

#### Deterministic Results

Over a 25-year time horizon, the average total costs were Can $97,497 (US $73,547.84) for the comparator group and Can $102,508 (US $77,327.93) for patients using *Medly*. The average total QALYs gained were 4.95 and 5.51 for the comparator group and *Medly* patients, respectively. When comparing the 2 groups, there was an incremental cost of Can $5011 (US $3780.10) with an incremental QALY gained of 0.566. This resulted in an ICER of Can $8850 (US $6676.09)/QALY (Table 5).

**Table 5:** Deterministic results of the reference case analysis.

Reference	Costs, Can $ (US $)	MCSE^a^, Can $ (US $)	QALYs^b^	MCSE	Incremental cost, Can $ (US $)	MCSE, Can $ (US $)	Incremental QALYs	MCSE	ICER^c^, Can $ (US $; $/QALY)
Comparator	97,497 (73,831)	3948 (2989)	4.95	0.12	N/A^d^	N/A	N/A	N/A	N/A
Medly	102,508 (77,626)	3592 (2720)	5.51	0.13	5011 (3794)	2014 (1525)	0.57	0.05	8850 (6701)

^a^MCSE: Monte Carlo standard error.

^b^QALY: quality-adjusted life year.

^c^ICER: incremental cost-effectiveness ratio.

^d^N/A: not applicable.

#### Probabilistic Results

**Figure 3:**
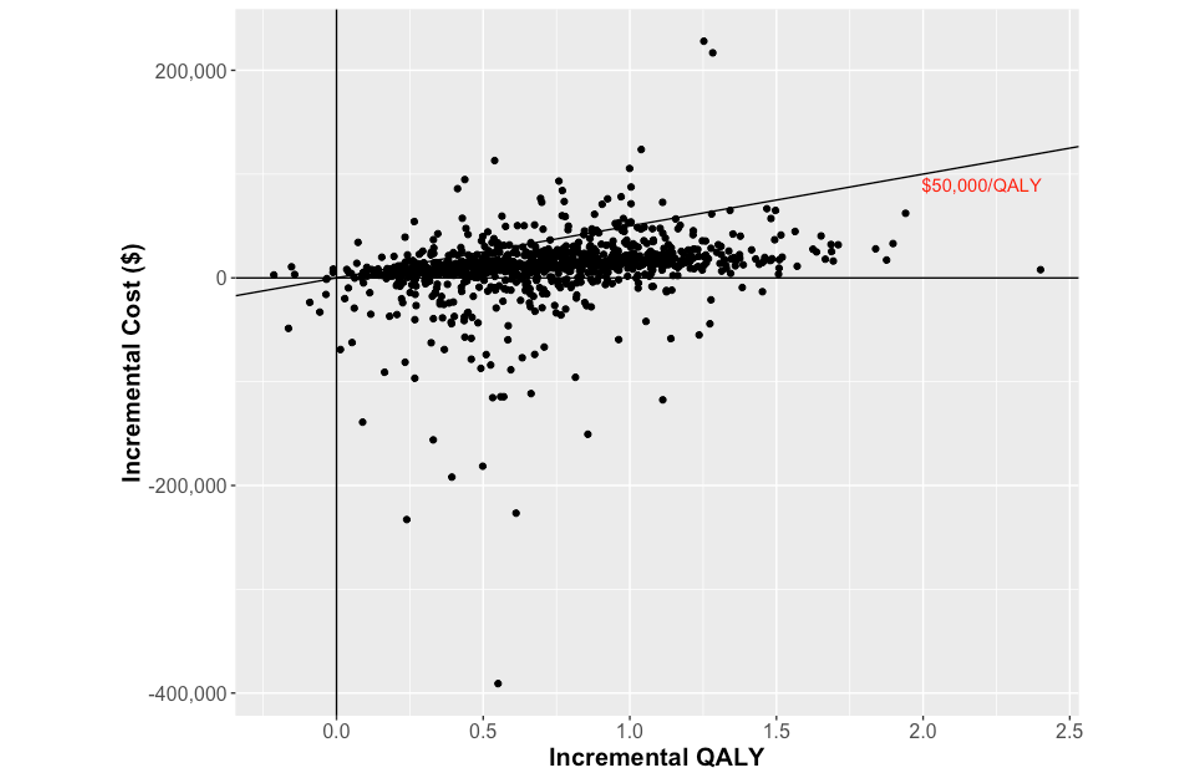
Cost-utility plane of 1000 iterations from the reference case probabilistic analysis. QALY: quality-adjusted life years.

**Figure 4:**
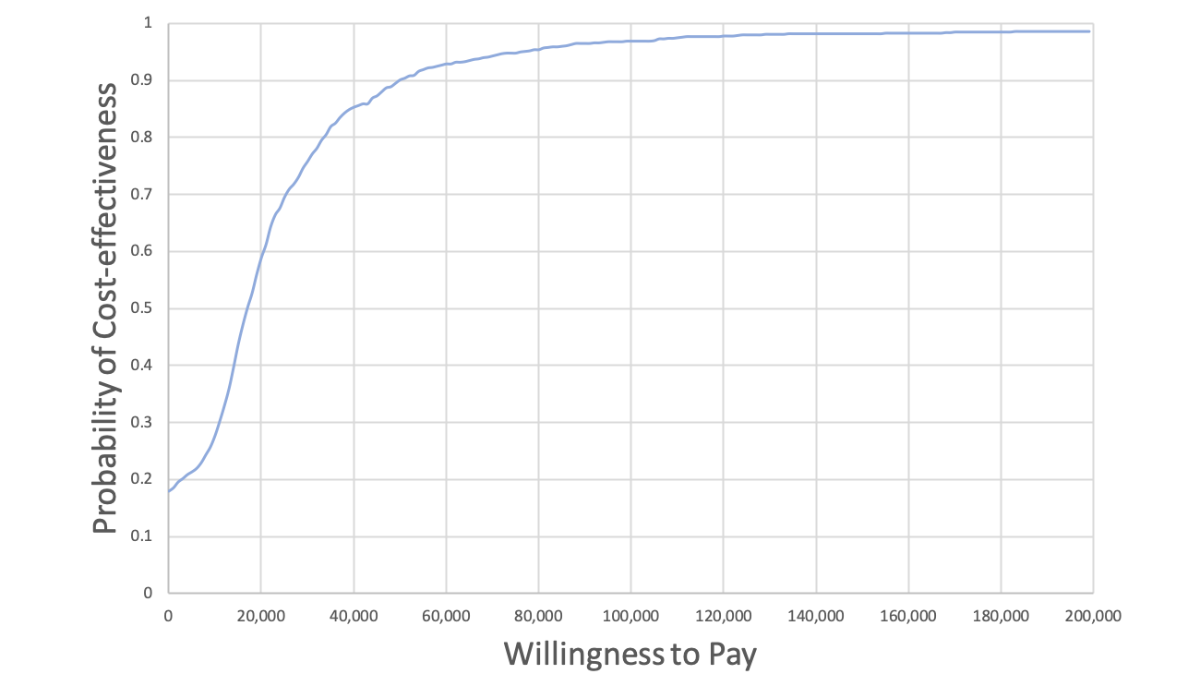
Cost-effectiveness acceptability curve of reference case probabilistic analysis.

On the basis of 1000 simulations of the reference case scenario in which each parameter was sampled from their respective distribution, 81.6% (816/1000) of the simulations showed that *Medly* was costlier and more effective, whereas 17.3% (173/1000) showed that *Medly* was less costly and more effective (Figure 3). The CEAC in Figure 4 shows that 90.1% (901/1000) of the simulations resulted in an ICER below the Can $50,000 (US $37,718)/QALY threshold.

### Scenario Analyses

#### NYHA Functional Classes

Table 6 presents the results of the NYHA functional class scenario analyses. As NYHA functional class increased, the average total costs and incremental costs increased. In addition, as NYHA functional class increased, total QALYs per population decreased. This led to a decreasing trend in ICERs with increasing NYHA functional class.

**Table 6:** Deterministic results for New York Health Association classes I, II, and III.

NYHA^a^ classes		Costs, Can $ (US $)	MCSE^b^, Can $ (US $)	QALYs^c^	MCSE	Incremental cost, Can $ (US $)	MCSE, Can $ (US $)	Incremental QALYs	MCSE	ICER^d^, Can $ (US $; $/QALY)
**NYHA I**										
	Comparator	81,714 (61,641)	3417 (2577)	6.89	0.13	N/A^e^	N/A	N/A	N/A	N/A
	Medly	88,016 (66395)	3215 (2425)	7.48	0.14	6302 (4753)	1806 (1362)	0.60	0.05	10,567 (7971)
**NYHA II**										
	Comparator	88,405 (66,689)	3821 (2882)	5.65	0.12	N/A	N/A	N/A	N/A	N/A
	Medly	94,335 (71,162)	3521 (2656)	6.35	0.13	5930 (4473)	2014 (1519)	0.70	0.06	8510 (6419)
**NYHA III**										
	Comparator	104,421	4356	4.12	0.11	N/A	N/A	N/A	N/A	N/A
	Medly	107,803	3929	4.69	0.11	3382	2134	0.57	0.05	5931

^a^NYHA: New York Health Association.

^b^MCSE: Monte Carlo standard error.

^c^QALY: quality-adjusted life year.

^d^ICER: incremental cost-effectiveness ratio.

^e^N/A: not applicable.

**Figure 5:**
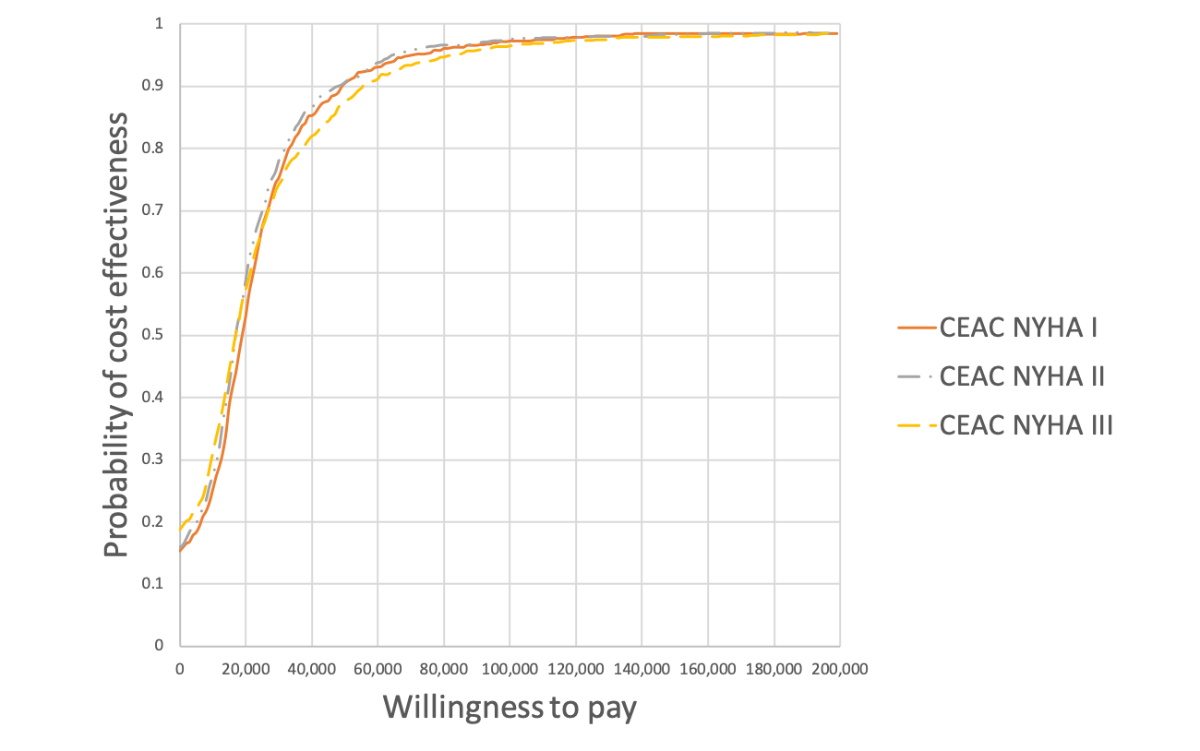
Cost-effectiveness acceptability curve of the New York Health Association functional class scenario analyses. CEAC: cost-effectiveness acceptability curve; NYHA: New York Health Association.

The CEAC curves for NYHA functional classes I, II, and III are shown in Figure 5. At a WTP threshold of $50,000 (US $37,718), the probability of cost-effectiveness for NYHA functional classes I, II, and III was 90.5% (905/1000), 90.6% (906/1000), and 87.5% (875/1000), respectively.

#### Deployment Models

Table 7 shows the results of the deployment model scenario analyses. As the only difference between scenarios was the total cost incurred by the *Medly* group, all comparator groups had the same average total costs and average total QALYs. The average total QALYs for patients using *Medly* were also the same for all scenarios. Moreover, as expected, when the proportion of FK users increased, so did the average total costs for patients using Medly. This led to ICERs following the same trend.

**Figure 6:**
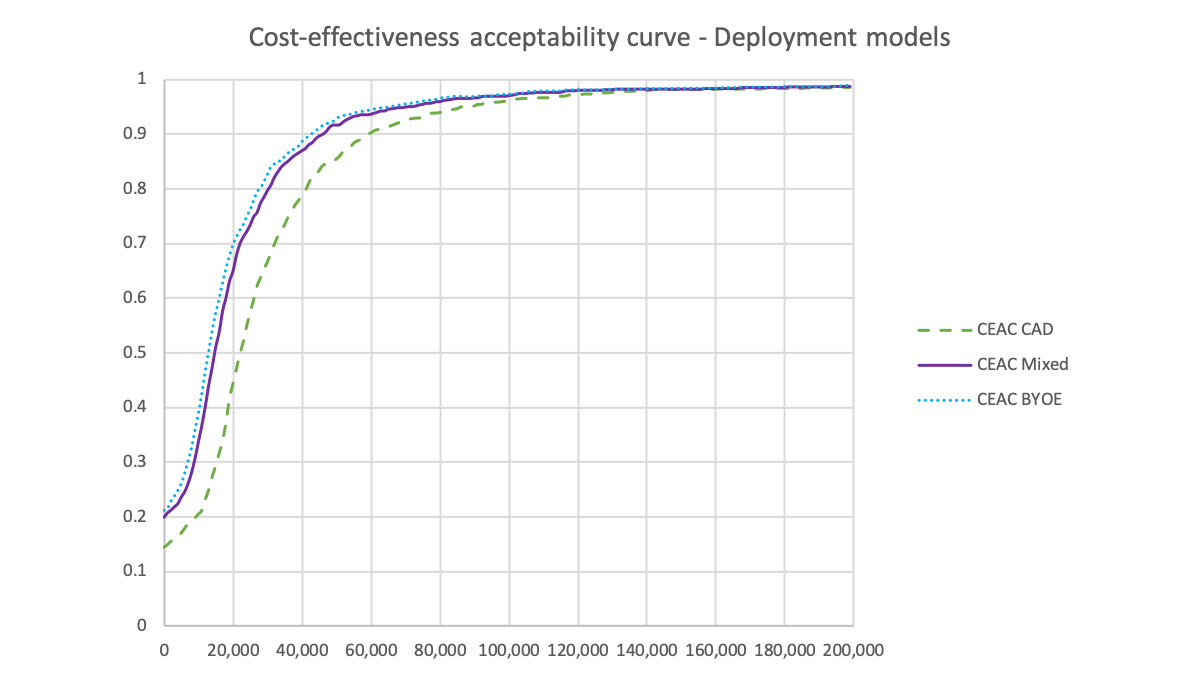
Cost-effectiveness acceptability curves for each deployment model in the scenario analyses. BYOE: Bring Your Own Everything; CEAC: cost-effectiveness acceptability curve; FK: Full Kit.

The CEAC curves for each deployment model are shown in Figure 6. At a WTP threshold of Can $50,000 (US $37,718), the probability of cost-effectiveness for all BYOE, mixed model, and all FK was 92.9% (929/1000), 91.7% (917/1000), and 85.4% (854/1000), respectively.

**Table 7:** Deterministic results for each deployment model of Medly.

Deployment models		Costs (Can $)	MCSE^a^ (Can $)	QALYs^b^	MCSE	Incremental cost (Can $)	MCSE (Can $)	Incremental QALYs	MCSE	ICER^c^ (Can $/QALY)
**All Bring Your Own Entertainment**										
	Comparator	97,497	3948	4.95	0.12	N/A^d^	N/A	N/A	N/A	N/A
	Medly	99,393	3542	5.51	0.13	1896	2006	0.57	0.05	3349
**Mixed model**										
	Comparator	97,497	3948	4.947	0.12	N/A	N/A	N/A	N/A	N/A
	Medly	100,769	3567	5.51	0.13	3273	2007	0.57	0.05	5780
**All Full Kit**										
	Comparator	97,497	3948	4.947	0.12	N/A	N/A	N/A	N/A	N/A
	Medly	106,194	3646	5.51	0.13	8697	2015	0.57	0.05	15,362

^a^MCSE: Monte Carlo standard error.

^b^QALY: quality-adjusted life year.

^c^ICER: incremental cost-effectiveness ratio.

^d^N/A: not applicable.

### Effectiveness Uncertainty

When the RR for mortality was set to its lower range, the ICER increased to Can $18,556 (US $13,997.90)/QALY (Table 8). When the RR for mortality was set to its upper range, *Medly* became dominant (Table 8). When the RR for hospitalization was set to its lower range, *Medly* became dominant. When the RR for hospitalization was set to its upper range, the ICER increased to Can $29,240 (US $22,057.49)/QALY.

**Table 8:** Deterministic results for the upper and lower limits of effectiveness in reducing mortality and hospitalization rates.

Effectiveness			Costs (Can $)	MCSE^a^ (Can $)	QALYs^b^	MCSE	Incremental cost (Can $)	MCSE (Can $)	Incremental QALYs	MCSE	ICER^c^ (Can $/QALY)
**RR^d^ for mortality**											
	**RR=0.70**										
		Comparator	97,497	3948	4.95	0.12	—^e^	—	—	—	—
		Medly	114,682	3995	5.87	0.13	17,186	2660	0.93	0.07	18,556
	**RR=0.94**										
		Comparator	97,497	3948	4.95	0.12	—	—	—	—	—
		Medly	91,542	3410	5.14	0.12	−5955	1339	0.19	0.03	−30,806
**RR for hospitalization**											
	**RR=0.63**										
		Comparator	97,497	3948	4.95	0.12	—	—	—	—	—
		Medly	92,107	3176	5.55	0.13	−5390	2126	0.61	0.06	−8895
	**RR=0.88**										
		Comparator	97,497	3948	4.95	0.12	—	—	—	—	—
		Medly	113,763	4052	5.50	0.13	16,267	2104	0.56	0.028	29,240

^a^MCSE: Monte Carlo standard error.

^b^QALY: quality-adjusted life year.

^c^ICER: incremental cost-effectiveness ratio.

^d^RR: relative risk.

^e^N/A: not applicable.

## Discussion

### Principal Findings

The purpose of this study was to assess the cost utility of the Medly program for patients with HF compared with the standard of care from a public payer perspective. The analysis showed that Medly had a high probability (90.1%) of being cost-effective at a WTP threshold of Can $50,000 (US $37,718)/QALY. The results also showed that cost-effectiveness improved in cohorts with more advanced HF. This is attributable to the higher health care utilization rates experienced in higher NYHA functional classes. Deployment models with larger proportions of patients bringing their own equipment to the *Medly* program were also shown to be more cost-effective owing to lower costs incurred by the program itself. Furthermore, the model itself was sensitive to the effectiveness parameters that informed the magnitude of the decrease in all-cause hospitalizations and mortality. However, the results of these analyses showed that *Medly* remains cost-effective even in scenarios with smaller clinical benefit, assuming a WTP threshold of Can $50,000 (US $37,718)/QALY [79,80].

### Study Implications

The significance of the study findings are 3-fold: (1) providing evidence for health care decision makers on the use of TM for HF, (2) supporting the use of a nurse-led model of TM using clinically validated algorithms within HF clinics, and (3) informing the use of economic modeling for future evaluation of early-stage health informatics technology.

Our study provided the *Medly* program with its first evaluation, where an economic perspective was considered. This added to the growing body of evidence associated with the program’s value not only for patients and health care professionals but also for the health care system. As discussions about implementing the *Medly* program at other sites in Ontario continue among decision makers and stakeholders, this study directly contributes to their understanding of *Medly’*s cost-effectiveness. It enables a new perspective on the upfront costs involved with implementing the TM infrastructure and purchasing necessary equipment, relative to the total costs a patient with HF incurs over a lifetime. Such evidence alleviates some of the uncertainty around the risks in introducing a new model of care for patients with HF.

A key factor contributing to the cost-effectiveness of Medly was the high number of patients (500) that a single nurse could manage, which is possible owing to the clinically validated algorithms that generate automatic clinical alerts and self-care messages. Other studies have reported a concern regarding increased clinical workload associated with incompatibility of the TM program with existing workflows, including management of and responding to alerts [81-83]. To mitigate the increased physician workload, the *Medly* program relies on a registered nurse coordinator and a rule-based algorithm [46] as a patient’s first point of contact, decreasing dependency on cardiologists. The registered nurse had the necessary skills to manage patient concerns and involved cardiologists as required. This nurse-led strategy presents a model of care that could be scalable to other hospitals.

Our study provides a case study on the use of multiple data sources and methods to develop a decision model for an early-stage health informatics intervention where knowledge gaps existed. As the purpose of this study was to evaluate the potential long-term effects of the *Medly* program in the management of patients with HF, the use of various data sources and modeling techniques are indispensable. This study was successful in developing a flexible algorithm based on the Cholesky decomposition method that was able to generate representative hypothetical cohorts of patients with HF according to the needs of the analysis [56]. An example of the algorithm’s flexibility to adapt to the needs of the analyses was the ability to generate hypothetical patient cohorts for NYHA classes I, II, and III while maintaining the individual differences between patients within each class. Our study also successfully implemented a highly validated multivariate Cox model, the SHFM [42-45], within our algorithm to predict the survival of each generated patient over their lifetime. As the purpose of this study was to understand the *long-term effects of the Medly* program, the inclusion of the most validated predictive model for HF survival was logical [84]. The use of the SHFM provided a link for the survival probabilities derived in our model to a larger body HF research around predictive modeling. As mentioned, this was similar to the study by Reed et al (2015) [42], in which the SHFM was used as its underlying prognosis model and correlated health care costs and utility values via regression techniques [42,85,86].

### Comparison With Other Work

Other studies have investigated the cost-effectiveness of other types of TM in HF, where data are transmitted to medical staff. The study by Thokala et al (2013) [38] compared TM with usual care from the public payer perspective and found TM to be cost-effective at £11,873/QALY gained in 2011 (equivalent to Can $19,996 (US $15,084.18)/QALY gained in 2018) using a two-state (alive or dead) cohort-based Markov model over a 30-year time horizon. This relatively higher ICER than ICER in our study, which could be attributed to various factors, such as the lower cost of hospitalizations (£1529.97-£2514.49), led to less cost-saving per hospitalization. Furthermore, model structures differed between the study by Thokala et al [38] and our study, in which different health states and transition probabilities were used. A study by Liu et al (2016) [40] also broadly compared TM with standard care from the payer perspective in the United States and found cost-savings for specific scenarios. This included patients who were intermediate and high-risk over 1- to 5-year time horizons. These results differ from our study owing to different model structures and transition probability parameters. The health states in the model developed by Liu et al (2016) were based on the number of past hospitalizations. In addition, hospitalization rates were conditional on both NYHA functional class and number of past hospitalizations, where the associated monthly probabilities for hospitalizations were much higher than those used in our study. This increased rate of hospitalizations in combination with the larger treatment effect size in reducing mortality and hospitalizations can be attributed to the cost-saving results [40]. A study by Grustam et al (2018) [39] compared TM with usual care for patients with HF from the public payer perspective within the Trans-European Network-Home-Care Management System using data from its original publication and other sources. This resulted in an ICER of €12,479/QALY gained in 2015 (Can $18,145 [US $13,687.86] in 2018), which was relatively higher than that reported in our study. The difference in our results can be attributed to the different methods used to model HF and measure effectiveness. The effectiveness of TM in the study was not defined by the risk for all-cause mortality and hospitalization events. Rather, effectiveness was measured by the decrease in probabilities of transitioning to more severe NYHA functional classes and the dead state based on an extrapolation from their database of patients with HF using TM [39].

### Limitations

As with any modeling exercise, it is important to understand the limitations around data availability and assumptions. First, owing to the lack of long-term studies, the trajectory of the effectiveness of TM was unknown and was assumed constant over the patient’s lifetime. It is not known if effectiveness changes over time, which may affect the results of this study. Another limitation was the assumption that patients used *Medly* over the entirety of the model. It has been reported that clinicians have not established a generalizable duration of enrollment into the program [87]. Patients may be enrolled into the program for a period of time and be off-boarded after they have learned the necessary self-care behaviors for HF and no longer require the assistance of the technology. This could decrease the costs of the intervention. In addition, caution should be exercised when interpreting data from the *Medly* program evaluation because patients are enrolled in the program based on a joint decision between the clinician and the patient, which could lead to selection bias. Without strict enrollment criteria and end time points, patients may be less sick than the average patients with HF, making results less generalizable. Another limitation was that it was assumed that the *QoL of patients using Medly* was same as that of patients in the comparator group. However, evidence from the *Medly* program evaluation [47] and past literature [88] indicates that QoL improves when patients use *Medly*. As QoL in these studies was measured using HF-specific scoring tools, translation of the improved QoL to utility values used in this study was not feasible because the QALY is derived from the EuroQol-5 Dimension instrument [89]. This likely underestimated *Medly*’s total QALYs gained. In addition, health care utilization data used to inform parameters in this study were based on the *Medly* program evaluation, which relied on a relatively small sample of patients, self-reported ED and GP visits, and a database that was limited to events that occurred at 1 hospital. This early-stage evidence on baseline health care utilization in patients with HF may underestimate or overestimate the actual health care utilization of an HF population, which could alter the results of the study.

### Conclusions

The *Medly* program was found to be a cost-effective solution given the widely cited WTP thresholds of Can $50,000 (US $37,718)/QALY for patients with HF when implemented within a multidisciplinary HF clinic in Ontario. This is the first Canadian economic evaluation of TM for HF using a cost-utility approach, and one of the few studies to use a long-term time horizon. The significance of this study includes providing evidence for health care decision makers on the use of TM for HF, supporting the use of a nurse-led model of TM within HF clinics, and informing the use of economic modeling for future evaluations of early-stage health informatics technology. Given the substantial impact of HF on patients’ QoL and burden on health care resources, expanding access to TM programs may be an important mechanism to improve HF disease management and patient outcomes.

## Appendix

Multimedia Appendix 1 Additional methodology supplementary material.

## Abbreviations

BYOE: Bring Your Own Everything
BYOP: Bring Your Own Phone
CEAC: cost-effectiveness acceptability curve
CUA: cost-utility analysis
ED: emergency department
FK: Full Kit
GP: general practitioner
HF: heart failure
ICER: incremental cost-effectiveness ratio
NYHA: New York Health Association
QALY: quality-adjusted life year
QoL: quality of life
RR: relative risk
SHFM: Seattle Heart Failure Model
TM: telemonitoring
UHN: University Health Network
WTP: willingness-to-pay
