# Supplementary material for: Evaluation of a Heart Failure Telemonitoring Program Through a Microsimulation Model: Cost-Utility Analysis
Source: J Med Internet Res. 2020 Oct 6;22(10):e18917. doi: 10.2196/18917 (PMC7576467; doi:10.2196/18917)
Supplement: Multimedia Appendix 1 [file jmir_v22i10e18917_app1.docx]

**Supplementary Material 1**

**SEATTLE HEART FAIULURE MODEL (SHFM)**

The SHFM consists of eighteen independent variables that relate to clinical, pharmacological, device and laboratory data. These variables are inputted into a regression model to yield a score value. This score is applied to a survival function and returns a probability of survival for a given patient according to the specified year. The baseline survival function used to derive the output was based on the large PRAISE1 study (n=1125), which was also used to develop the SHFM. The survival function is:


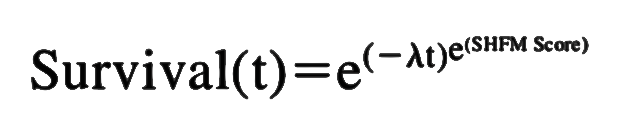


where t is the time of estimated survival, λ is a constant derived from PRAISE1 (λ=0.0405), and SMFH score is the output of the SHFM regression.

Since time passes over the simulation period, the time parameter can be used to adjust various transitions probabilities that would change over time. Specifically, the probability of mortality increases over time. To capture this increase, an updated transition probability for death is derived from the SHFM after each 1-month iteration. This value is based on the updated age value and NYHA functional class, and then extrapolated from the updated survival curve.

**Table A**. Beta coefficients used in the SHFM to calculate survival curves per patient. For the probabilistic analysis, a log normal distribution was applied to each coefficient. ρ represents the variable in the correlation matrix below.

| **Variable Description** | **ρ** | **Beta Coefficient (SD) from Levy et al. (2006)** |
| --- | --- | --- |
| Age (Decade) | 1 | 1.09 (1.053) |
| Gender | 2 | 1.089 (1.142) |
| NYHA class | 3 | 1.6 (1.259) |
| 100/(Ejection fraction) | 4 | 1.03 (1.010) |
| Ischemic etiology | 5 | 1.354 (1.125) |
| min(Systolic blood pressure, 160)/10 | 6 | 0.877 (1.033) |
| Diuretic dose/weight | 7 | 1.178 (1.037) |
| Allopurinol Use | 8 | 1.571 (1.162) |
| max(138-Sodium, 0) | 9 | 1.05 (1.023) |
| 100/Cholesterol | 10 | 2.206 (1.464) |
| max(16-Hemoglobin, 0) | 11 | 1.336 (1.034) |
| max(Hemoglobin-16, 0) | 12 | 1.124 (1.153) |
| min(% Lymphocytes, 47)/5 | 13 | 0.897 (1.030) |
| max(Uric acid, 3.4) | 14 | 1.064 (1.021) |
| ACE-inhibitor and/or ARB | 15 | 0.77 (1.074) |
| Beta blocker | 16 | 0.66 (1.068) |
| Aldosterone blocker | 17 | 0.76 (1.109) |
| Implantable Cardioverter-Defibrillator | 18 | 0.74 (1.052) |

**CHOLESKY DECOMPOSITION**

**Table B**. The correlation matrix (in tabular format) that was used for generating patient profiles.

|  | *Ρ,*1 | *ρ,*2 | *ρ,*3 | *ρ,*4 | *ρ,*5 | *ρ,*6 | *ρ,*7 | *ρ,*8 | *ρ,*9 | *ρ,*10 | *ρ,*11 | *ρ,*12 | *ρ,*13 | *ρ,*14 | *ρ,*15 | *ρ,*16 | *ρ,*17 | *ρ,*18 |
| --- | --- | --- | --- | --- | --- | --- | --- | --- | --- | --- | --- | --- | --- | --- | --- | --- | --- | --- |
| *ρ*1*,* | 1.000 | -0.056 | 0.238 | 0.040 | 0.118 | 0.190 | -0.165 | -0.044 | -0.042 | -0.083 | 0.020 | -0.173 | 0.140 | -0.048 | -0.146 | 0.056 | -0.280 | -0.021 |
| *ρ*2*,* | -0.056 | 1.000 | 0.157 | -0.016 | -0.059 | -0.056 | 0.033 | -0.001 | 0.012 | 0.070 | 0.033 | -0.059 | -0.036 | -0.108 | 0.280 | 0.112 | 0.321 | -0.006 |
| *ρ*3*,* | 0.238 | 0.157 | 1.000 | 0.016 | 0.073 | -0.019 | 0.006 | -0.023 | 0.004 | -0.027 | -0.011 | -0.080 | -0.032 | -0.062 | 0.001 | 0.045 | -0.034 | 0.007 |
| *ρ*4*,* | 0.040 | -0.016 | 0.016 | 1.000 | -0.334 | 0.236 | 0.127 | -0.033 | 0.009 | -0.143 | 0.033 | 0.061 | 0.102 | 0.036 | -0.019 | -0.204 | -0.011 | -0.196 |
| *ρ*5*,* | 0.118 | -0.059 | 0.073 | -0.334 | 1.000 | -0.158 | -0.225 | 0.023 | -0.202 | 0.051 | 0.076 | -0.066 | -0.090 | -0.049 | -0.058 | 0.268 | -0.010 | 0.287 |
| *ρ*6*,* | 0.190 | -0.056 | -0.019 | 0.236 | -0.158 | 1.000 | 0.009 | -0.081 | 0.016 | -0.043 | -0.016 | 0.069 | 0.205 | 0.134 | 0.065 | -0.140 | 0.029 | -0.177 |
| *ρ*7*,* | -0.165 | 0.033 | 0.006 | 0.127 | -0.225 | 0.009 | 1.000 | 0.044 | 0.077 | -0.035 | -0.017 | 0.069 | -0.010 | 0.007 | -0.009 | -0.053 | 0.086 | -0.101 |
| *ρ*8*,* | -0.044 | -0.001 | -0.023 | -0.033 | 0.023 | -0.081 | 0.044 | 1.000 | 0.029 | -0.037 | -0.015 | -0.061 | -0.145 | 0.031 | -0.042 | 0.032 | 0.012 | 0.083 |
| *ρ*9*,* | -0.042 | 0.012 | 0.004 | 0.009 | -0.202 | 0.016 | 0.077 | 0.029 | 1.000 | -0.077 | -0.131 | 0.006 | -0.021 | 0.017 | -0.024 | -0.040 | 0.032 | -0.055 |
| *ρ*10*,* | -0.083 | 0.070 | -0.027 | -0.143 | 0.051 | -0.043 | -0.035 | -0.037 | -0.077 | 1.000 | -0.012 | 0.052 | -0.011 | 0.025 | 0.062 | 0.051 | 0.072 | 0.020 |
| *ρ*11*,* | 0.020 | 0.033 | -0.011 | 0.033 | 0.076 | -0.016 | -0.017 | -0.015 | -0.131 | -0.012 | 1.000 | -0.039 | -0.007 | -0.058 | -0.001 | 0.029 | 0.032 | 0.097 |
| *ρ*12*,* | -0.173 | -0.059 | -0.080 | 0.061 | -0.066 | 0.069 | 0.069 | -0.061 | 0.006 | 0.052 | -0.039 | 1.000 | 0.101 | 0.126 | 0.130 | -0.115 | 0.048 | -0.155 |
| *ρ*13*,* | 0.140 | -0.036 | -0.032 | 0.102 | -0.090 | 0.205 | -0.010 | -0.145 | -0.021 | -0.011 | -0.007 | 0.101 | 1.000 | 0.002 | 0.018 | -0.076 | -0.003 | -0.158 |
| *ρ*14*,* | -0.048 | -0.108 | -0.062 | 0.036 | -0.049 | 0.134 | 0.007 | 0.031 | 0.017 | 0.025 | -0.058 | 0.126 | 0.002 | 1.000 | 0.175 | 0.005 | -0.013 | -0.037 |
| *ρ*15*,* | -0.146 | 0.280 | 0.001 | -0.019 | -0.058 | 0.065 | -0.009 | -0.042 | -0.024 | 0.062 | -0.001 | 0.130 | 0.018 | 0.175 | 1.000 | -0.002 | 0.194 | -0.084 |
| *ρ*16*,* | 0.056 | 0.112 | 0.045 | -0.204 | 0.268 | -0.140 | -0.053 | 0.032 | -0.040 | 0.051 | 0.029 | -0.115 | -0.076 | 0.005 | -0.002 | 1.000 | 0.122 | 0.349 |
| *ρ*17*,* | -0.280 | 0.321 | -0.034 | -0.011 | -0.010 | 0.029 | 0.086 | 0.012 | 0.032 | 0.072 | 0.032 | 0.048 | -0.003 | -0.013 | 0.194 | 0.122 | 1.000 | 0.108 |
| *ρ*18*,* | -0.021 | -0.006 | 0.007 | -0.196 | 0.287 | -0.177 | -0.101 | 0.083 | -0.055 | 0.020 | 0.097 | -0.155 | -0.158 | -0.037 | -0.084 | 0.349 | 0.108 | 1.000 |

**ALL-CAUSE HOSPITALIZATIONS**

Using data from the *Medly* Program Evaluation, the risk of all-cause hospitalization was compared using a pre-post approach, where the risk of hospitalization 6 months prior to baseline was compared to the risk at 6 months post-intervention. All-cause hospitalizations were used due to limitations in identifying HF-specific events stemming from the commonality of patients having comorbidities.(1) Some patients died post-intervention without being hospitalized. This could lead to underestimation of hospitalization . Thus, a conservative approach was taken for this analysis to account for this bias. A composite endpoint was used where it was assumed that patients who died would have been hospitalized. Table 5 shows the two-by-two table of patients who experienced a hospitalization 6 months prior to baseline and 6 months after baseline. The McNemar test was used to calculate the odds ratio, which is a non-parametric test for paired nominal data, which evaluates the magnitude of the difference between the discordant cells (i.e. number of patients who were not hospitalized 6 months prior to baseline to number of patients who were hospitalized 6 months after baseline).(2) The odds ratio was then converted to a relative risk, based on the following equation(3):

$$RR=\frac{OR}{\left( 1-P \right)+(P *OR)}$$

Where RR is the relative risk, OR is the odds ratio from McNemar’s test and P is the prevalence of the outcome in the reference group

Based on this, the relative risk (RR) used in the Markov model was 0.753 (0.634 – 0.879). For reference, this was comparable to 0.94 (0.85 – 1.03) RR reported by the meta-analysis conducted by Yun et al. (2018), which compared HF patients using TM to HF patients not using TM with study follow-up ranging from 3 months to 15 months, with one study having a 4 year follow-up.(4)

**Table C**. McNemar’s odds ratio for a hospitalization event prior to using *Medly* compared to the period when patients were using *Medly*.

|  | **Hospitalized or death** | **Not hospitalized** | **Total Number of Patients** | **McNemar’s Odd Ratio for paired data (95% CI)** | **p-value** |
| --- | --- | --- | --- | --- | --- |
| **6 months prior to baseline** | 147 | 104 | 315 |  |  |
| **6 months after baseline** | 168 | 211 | 315 | 0.619 (0.480 – 0.795) | <0.001 |

**ADJUSTING MORTALITY AND HOSPITALIZATION PROBABILITIES FOR TREATMENT EFFECTS**

Based on the evidence presented above, there was indication that the *Medly* Program should be effective in reducing mortality and hospitalization rates, though a statistically non-significant trend towards reduced hospitalizations was observed. Sensitivity analysis was conducted to explore this uncertainty. It was assumed that the magnitude of effect that *Medly* would have would be at least equivalent to those reported in literature. The transition probabilities specific to hospitalization and mortality within the model were imputed with this treatment effect. This was done by assuming a constant risk over time and by converting the transition probability into an instantaneous rate using the following equation:(5)

$Rate= -\frac{\left[ \ln\left( 1-probability \right) \right]}{time}$ **Equation 1**

With the instantaneous rate calculated, it was adjusted by a RR as follows:

$Adjusted Rate=Rate*RR$ **Equation 2**

Then, the adjusted rate can be converted back into a probability as follows:

$Adjusted Probability=1-exp(-adjusted rate*time)$ **Equation 3**

These equations were used to adjust all transition probabilities related to mortality and hospitalizations for each patient that entered the model when using *Medly*. Furthermore, it was assumed that the effect of TM was constant across patient characteristics.

**CURVE FITTING**

Table D. Akaike information criterion (AIC) and Bayesian information criterion (BIC) scores for each distribution fitted for the healthcare utilization data from the Medly Program Evaluation.

| GP Visits | Information Criteria Method | Geometric | Poisson | Negative Binomial | Size (se) | mu (se) |
| --- | --- | --- | --- | --- | --- | --- |
| Entire Cohort | AIC | 457.19 | 604.94 | 448.29 | 0.49 (0.10) | 1.58 (0.22) |
|  | BIC | 460.07 | 607.82 | 454.06 |  |  |
| NYHA I | AIC | 62.63 | 97.15 | 54.78 | 0.17 (0.10) | 1.20 (0.69) |
|  | BIC | 63.62 | 98.15 | 56.78 |  |  |
| NYHA II | AIC | 238.71 | 322.99 | 233.99 | 0.47 (0.13) | 1.57 (0.31) |
|  | BIC | 240.94 | 325.11 | 238.46 |  |  |
| NYHA III | AIC | 153.56 | 179.55 | 155.52 | 0.92 (0.37) | 1.85 (0.37) |
|  | BIC | 155.28 | 181.26 | 158.94 |  |  |
| OP Visits |  |  |  |  |  |  |
| Entire Cohort | AIC | 775.52 | 806.71 | 755.86 | 2.50 (0.56) | 2.46 (0.16) |
|  | BIC | 778.75 | 809.94 | 762.31 |  |  |
| NYHA I | AIC | 113.51 | 114.01 | 110.44 | 3.38 (2.20) | 2.65 (0.43) |
|  | BIC | 114.77 | 115.27 | 112.95 |  |  |
| NYHA II | AIC | 403.32 | 426.83 | 398.81 | 2.00 (0.60) | 2.20 (0.21) |
|  | BIC | 405.93 | 429.45 | 404.04 |  |  |
| NYHA III | AIC | 253.12 | 257.92 | 243.66 | 3.16 (1.26) | 2.84 (0.31) |
|  | BIC | 255.16 | 259.97 | 247.75 |  |  |
| ED Visits |  |  |  |  |  |  |
| Entire Cohort | AIC | 430.43 | 617.46 | 415.56 | 0.42 (0.09) | 1.21 (0.18) |
|  | BIC | 433.38 | 620.41 | 421.46 |  |  |
| NYHA I | AIC | 41.75 | 41.93 | 43.61 | 1.90 (4.05) | 0.45 (0.16) |
|  | BIC | 42.84 | 43.02 | 45.79 |  |  |
| NYHA II | AIC | 251.01 | 422.11 | 229.68 | 0.29 (0.07) | 1.50 (0.35) |
|  | BIC | 253.32 | 424.41 | 234.29 |  |  |
| NYHA III | AIC | 127.88 | 134.21 | 129.25 | 1.53 (0.90) | 1.12 (0.21) |
|  | BIC | 129.64 | 135.97 | 132.77 |  |  |

Geometric, Poisson and negative binomial distributions were fitted for GP visits, outpatient clinic visits and ED visits for the entire cohort and each NYHA class. AIC and BIC scores were used to choose distribution shapes. Both criteria are a measure of the relative quality of a statistical model by trading off the model fit with a number of model parameters – the lower the AIC and/or BIC, the better the model.(6) Since the majority of distributions indicated negative binomial as the best fit, including the best fit for the entire cohort for each healthcare service, negative binomial distributions were chosen for all NYHA classes.

References

1. Canadian Institute for Health Information. All-Cause Readmission to Acute Care and Return to the Emergency Department. 2012;64.

2. Fay MP. Exact McNemar’s Test and Matching Conﬁdence Intervals. R-Project. :6.

3. Zhang J, Yu KF. What’s the relative risk? A method of correcting the odds ratio in cohort studies of common outcomes. JAMA. 1998 Nov 18;280(19):1690–1.

4. Yun JE, Park J-E, Park H-Y, Lee H-Y, Park D-A. Comparative Effectiveness of Telemonitoring Versus Usual Care for Heart Failure: A Systematic Review and Meta-analysis. Journal of Cardiac Failure. 2018 Jan;24(1):19–28.

5. Fleurence RL, Hollenbeak CS. Rates and Probabilities in Economic Modelling. Pharmacoeconomics. 2007 Jan 1;25(1):3–6.

6. Jackson CH, Thompson SG, Sharples LD. Accounting for uncertainty in health economic decision models by using model averaging. Journal of the Royal Statistical Society: Series A (Statistics in Society). 2009 Apr 1;172(2):383–404.
